# A Giant Cavernous Hemangioma of the Left Atrioventricular Groove

**DOI:** 10.1155/2017/6898629

**Published:** 2017-03-20

**Authors:** Chengming Fan, Changming Tan, Demiao Kong, Jinfu Yang, Shuwen Yuan, Sijie Wu

**Affiliations:** ^1^Department of the Cardiovascular Surgery, The Second Xiangya Hospital, Central South University, Middle Renmin Road 139, Changsha 410011, China; ^2^Hunan Provincial Key Laboratory of Cardiovascular Research, Changsha 410078, China; ^3^Department of Radiology, The Second Xiangya Hospital, Central South University, Changsha, China

## Abstract

A 10-year-old Chinese female diagnosed with an asymptomatic giant cardiac cavernous hemangioma was reported. The patient originally tended to observation because this unusual cardiac tumoral mass was discovered incidentally during routine health examination of transthoracic echocardiography. Over 5 years of follow-up, the mass had enlarged obviously, and the patient visited our outpatient clinic and was prone to excision. Subsequently, a total resection surgery of the tumor was performed, and the tumor was found to be located on the left atrioventricular groove with complete packing membrane. The patient was discharged on postoperative day 4 and remains asymptomatic on last follow-up.

## 1. Introduction

Cardiac primary tumors are extremely rare with prevalence of 0.0017 to 0.28% in autopsy findings. Myxomas are the most frequent cardiac tumor (75%), while cardiac hemangiomas are uncommon and are found in 1-2% of cases [1]. However, improvements in noninvasive detection of cardiac tumors have led to an increase in the frequency of this diagnosis [2]. In this report, we present a case of a 15-year-old female, free of traditional cardiac risk factors, who required surgery removal because of the enlargement of the mass during the observational follow-up after the first detection during routine health examination of transthoracic echocardiography 5 years earlier.

## 2. Case Report

**Figure 1:**
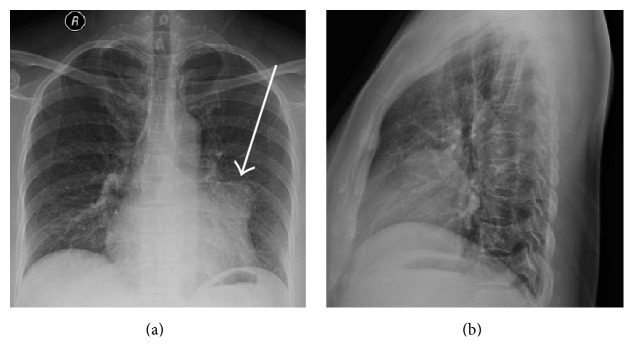
Chest radiography showed small opacity in the left lower lobe (arrow).

**Figure 2:**
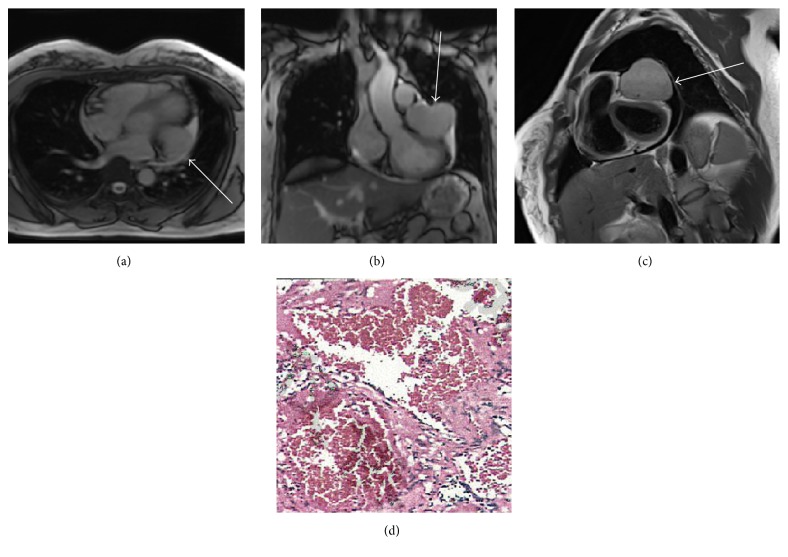
Cardiac magnetic resonance imaging showed a hypointense tumorous mass ((a), (b), and (c) arrows) extending from the internal wall of pericardium towards the adjacent wall of the left ventricle with a clear adipose line between the mass and the left ventricle. Pathology (d) showed cavernous hemangioma with some myocardial tissue denaturation, partial cardiac collagen degeneration, hypertrophy, and enlargement of the nucleus and immunostaining showed positive Ki-67 and negative SMA. Hematoxylin and eosin staining. Magnification, ×100.

A 10-year-old girl was referred to our cardiovascular department to perform an assessment of a cardiac mass detected during routine health examination of transthoracic echocardiography. The patient initially declined observation. On presentation to our institution 5 years later, the lesion had enlarged obviously, and the patient consented to remove the unusual cardiac mass. Chest radiography showed small opacity in the left lower lobe (Figures 1(a) and 1(b), arrow). Cardiac magnetic resonance was obtained with four-chamber (Figure 2(a)) and coronal (Figure 2(b)) view depicting a hypointense tumorous mass (48 mm × 57 mm) extending from the internal wall of pericardium towards the adjacent wall of the left ventricle with the left ventricle being squashed severely (arrow). T1-weighted sequence demonstrated a clear adipose line between the mass and the left ventricle. Cardiac cine magnetic resonance imaging demonstrated a clear boundary of the mass and the ventricular ejection fraction was 49% (Figure 2(c)). At surgery, a median sternotomy approach was used, and the pericardium was opened. The mass was found to be located in the left atrioventricular groove and was densely connected to the left atrial appendage. Consequently, a total resection of the mass together with part of the left atrial appendage was performed and pathology showed cavernous hemangioma with some myocardial tissue denaturation, partial cardiac collagen degeneration, hypertrophy, and enlargement of the nucleus (Figure 2(d)); immunostaining showed positive Ki-67 and negative SMA. The patient was discharged home in stable condition on postoperative day 4 and showed no evidence of recurrence on 16-month follow-up.

## 3. Discussion

Cardiac hemangioma is a rare primary benign tumor which accounts for only 2% of primarily resected cardiac tumors [3]. Cardiac hemangioma could locate in any part of the heart, with predominance of ventricular septum and right atrium. Most of the patients were asymptomatic, and the tumors were incidental autopsy findings (<1%) [1]. Although most cardiac neoplasms are incidentally discovered, these lesions have the potential to cause variety of symptoms secondary to their spatial relation to adjacent structures. Presentations include arrhythmias, chest pain, heart failure, cough, dysphagia, and coronary insufficiency. As echocardiographic techniques continue to improve, cardiac tumors are being detected earlier, often before significant symptoms have developed. This increasingly poses a challenge to cardiologists and surgeons, because the lack of associated clinical indicators makes it more difficult to distinguish patients who need surgery from those who will benefit from conservative follow-up [4]. Pathology still serves as a gold standard of the diagnosis of cardiac cavernous hemangioma. The natural history for cardiac hemangiomas is unpredictable. Spontaneous regression of a cardiac cavernous hemangioma has been reported [5]. However, surgical excision should be performed as early as possible, especially in cases with large cardiac tumors, increasing size, symptoms, and an unclear diagnosis [6]. In our case, a total resection was performed because of the enlargement of the mass during the observational follow-up after the first detection during routine health examination of transthoracic echocardiography 5 years earlier.

## 4. Conclusion

In conclusion, we report a very rare case of cardiac hemangioma located on the left atrioventricular groove. Surgical excision of tumor mass had a favorable outcome. The patient was well and had no recurrence 16 months after surgery.
